# LncRNA FAM83A‐AS1 promotes lung adenocarcinoma progression by enhancing the pre‐mRNA stability of FAM83A

**DOI:** 10.1111/1759-7714.13928

**Published:** 2021-03-09

**Authors:** Wenyi Wang, Zhunlin Zhao, Chun Xu, Chang Li, Cheng Ding, Jun Chen, Tengfei Chen, Jun Zhao

**Affiliations:** ^1^ Department of Thoracic Surgery, The First Affiliated Hospital of Soochow University Medical College of Soochow University Suzhou China

**Keywords:** FAM83A, FAM83A‐AS1, lncRNA, LUAD

## Abstract

**Background:**

Lung cancer is the leading cause of cancer deaths worldwide. Long non‐coding RNAs (lncRNAs) affect a series of cellular biological processes, including oncogene function promotion. In this study, we explored the functions and mechanisms of FAM83A antisense RNA 1 (FAM83A‐AS1) in non‐small cell lung cancer (NSCLC) progression.

**Methods:**

The expression of FAM83A‐AS1and FAM83A mRNA was analyzed using the Cancer Genome Atlas (TCGA) data. Proliferation, migration, invasion and Western blotting were measured after treatment with overexpressed or knockdown FAM83A‐AS1. To determine the relationship between FAM83A‐AS1 and FAM83A, RNase protection assay (RPA), amanitin treatment, RNA pulldown assay and RNA immunoprecipitation (RIP) assay were performed.

**Results:**

High expression of FAM83A‐AS1 in lung adenocarcinoma (LUAD) was closely associated with low overall survival (OS) and progression‐free survival (PFS). Functionally, high FAM83A‐AS1 expression increased LUAD cell proliferation and metastasis, indicating that FAM83A‐AS1 exerted its oncogenic functions. Furthermore, FAM83A‐AS1 promoted NSCLC progression via ERK signaling pathways. Mechanistically, FAM83A‐AS1 post‐transcriptionally increased FAM83A expression by enhancing pre‐mRNA stability. FAM83A‐AS1 enhanced FAM83A mRNA stability not only by forming an RNA duplex but also by binding to FBL.

**Conclusions:**

We determined that FAM83A‐AS1 increased FAM83A expression by enhancing FAM83A pre‐mRNA stability and promoted the tumorigenesis of LUAD.

## Graphical abstract

High expression of FAM83A‐AS1 in lung adenocarcinoma (LUAD) was closely associated with low overall survival (OS) and progression‐free survival (PFS). FAM83A‐AS1 increased FAM83A expression by enhancing FAM83A pre‐mRNA stability and promoted the tumorigenesis of LUAD.

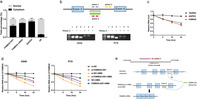

## INTRODUCTION

Lung cancer is the leading cause of cancer deaths in both men and women worldwide.^1^ Non‐small cell lung cancer (NSCLC) accounts for approximately 85% of all lung cancer cases. NSCLC can be classified into two histological subtypes: adenocarcinoma (ADE) and squamous cell carcinoma (SCC).^2^ Despite many advances made regarding treatment strategies for NSCLC, the overall five‐year survival rate is still meager.^3^ Therefore, there is an urgent need to discover novel molecular targets against NSCLC.

Long non‐coding RNAs (lncRNAs) are non‐coding RNAs containing over 200 nucleotides.^4^ In recent years, extensive evidence has suggested that lncRNAs are involved in the occurrence of many diseases, including cancer.^5^, ^6^ lncRNAs affects a series of cellular biological processes including cell proliferation, apoptosis resistance, angiogenesis, and metastasis,^7^ and participate in tumor progression mainly by promoting oncogene function or by inhibiting tumor suppressor genes.

FAM83A antisense RNA 1 (FAM83A‐AS1) is composed of 1572 nucleotides. It is transcribed from the antisense strand of the FAM83A gene located at 8q24.13. FAM83A is an oncogene that is upregulated in many cancers.^8^, ^9^ There is a complementary region in the intron between exon 3 and exon 4 of the FAM83A gene. FAM83A‐AS1 has been reported to play an essential role in the development and progression of NSCLC and hepatocellular carcinoma.^10^, ^11^ Nevertheless, the molecular role of FAM83A‐AS1 in the development of NSCLC has not as yet been fully explored.

In this study, we found that FAM83A‐AS1 was upregulated in lung adenocarcinoma (LUAD) and was associated with overall survival (OS) and progression‐free survival (PFS). Subsequently, we elucidated the potential mechanism by which FAM‐AS1 promotes LUAD progression.

## METHODS

### Cell culture

NSCLC cell lines (HBE, A549, H460, H1650 and PC9) were cultured in RPMI‐1640 medium containing 10% fetal bovine serum (FBS) at 37°C in a humidified 5% CO_2_ incubator. All NSCLC cell lines were purchased from the Chinese Academy of Sciences (Shanghai, China).

### Quantitative reverse transcription‐PCR (qRT‐PCR)

Total RNA extraction, cDNA synthesis, and quantitative real‐time polymerase chain reaction (qRT‐PCR) analysis were performed as previously described.^12^ In brief, cell RNA was extracted using TRIzol reagent (Invitrogen Life Technologies), following the manufacturer's instructions. cDNA was synthesized using a cDNA Reverse Transcription kit (Applied Biosystems). The qRT‐PCR analysis was performed using an SYBR Green kit (Invitrogen Life Technologies) in an ABI 7900 system (Applied Biosystems). The RNA expression levels were normalized to the GAPDH level. The primers used were synthesized by GENEWIZ.

### Plasmid and siRNA transfection

The full‐length sequence of FAM83A‐AS1 was synthesized and subcloned to the pcDNA3.1 vector by GENEWIZ (Suzhou). NSCLC cells were transfected with the siRNA (Genepharma) at a final concentration of 150 nmol/l on a Lipofectamine 2000 system (Invitrogen). The siRNA target sequences were as follows: si‐NC 5′‐CCCATAAGAGTAATAATAT‐3′; si‐FAM83A‐AS1‐1 (si‐1) 5′‐AGAGTAAGCAAGATAGAGAC‐3′; si‐FAM83A‐AS1‐2 (si‐2) 5′‐AGGCTAGTAAGCAGGTCACC‐3′; si‐FAM83A‐1 5′‐GCCGCCTTAGCAGCAGCAGT‐3′; and si‐FAM83A‐2 5′‐CCGCCTTAGCAGCAGCAGTG‐3′.

### Cell counting Kit‐8 (CCK‐8) assay

Cell viability was determined using a CCK‐8 assay. After treatment for 24 h, NSCLC cells were seeded into 96‐well plates (3000 cells/well). The cells were incubated with a CCK‐8 reagent (10 μl/well; Beijing Solarbio) at 37°C for 1 h, and optical density (OD) was measured absorbance at 450 nm.

### RNase protection assay (RPA)

RPA was performed, as previously reported.^13^ Total RNA was extracted using TRIZOL reagent (Invitrogen). Then, the RNA samples were treated using RNase A (Tiangen) and incubated at 37°C for 30 min. RT‐PCR was used to detect duplex formation within the overlapping region or nonoverlapping regions.

### Amanitin treatment

A total of 2 × 10^6^ LUAD cells were treated with α‐amanitin (10 μg/ml, Sigma‐Aldrich) for different durations (0, 6, 12, and 24 h). mRNA was then extracted and detected through qRT‐PCR. The 18S gene is not affected by α‐amanitin and was used as an internal control.

### Cell nucleus/cytoplasm fraction isolation

The separation of the cytoplasm from the nucleus fraction was performed using a nuclear and cytoplasmic extraction kit (Ambion), following the manufacturer's instructions. Cytoplasmic and nuclear RNA was isolated using TRIzol reagent (Thermo Fisher Scientific).

### Transwell migration and invasion assay

For the transwell migration assay, 5 × 10^4^ NSCLC cells in serum‐free medium were plated into the upper chamber (Corning, USA), while 600 μl of medium containing 10% FBS was added into the lower chamber. After 24 h of incubation, the cells were fixed with 4% formaldehyde and stained with 0.1% crystal violet (Beyotime). Cells still in the upper chamber were removed using cotton swabs. The cells that had migrated were photographed and counted under an inverted fluorescence microscope (Nikon). The invasion assay was conducted in the same manner, except that the upper chamber membrane was coated with Matrigel, and the cells were plated into the chamber and incubated for 48 h.

### Western blotting (WB) analysis

The cells were lysed using a buffer (Beyotime, China) with a phosphatase and protease inhibitor cocktail (Beyotime, China). The proteins were separated using SDS‐polyacrylamide gel electrophoresis (PAGE) and then transferred onto a PVDF membrane (Millipore, USA). The blots were blocked using 5% skimmed milk and were incubated with antibodies against GAPDH (AM1020a, Abgent), FBL (66985‐1‐Ig, Proteintech), FAM83A (ab128245, Abcam), E‐cadherin (610 181, BD), p‐ERK (# 4370S, CST), and ERK (# 9107S, CST). After washing, the membranes were incubated with (HRP)‐conjugated secondary antibodies. The protein expression levels were visualized using an ECL detection system (Tanon, China).

### RNA pulldown assay

The RNA pulldown assay was performed using an RNA‐protein pulldown kit (Thermo Scientific Pierce). FAM83A‐AS1 and FAM83A‐AS1 antisense sequences (negative control) were cloned into pcDNA‐3.1 vectors. The RNAs were transcribed using T7 RNA polymerase (Promega) in vitro and were labeled with biotin. The labeled RNAs were then incubated with streptavidin magnetic beads and mixed with cell extracts overnight at 4°C. The RNA‐protein complex was detected using Western blotting analysis.

### RNA immunoprecipitation (RIP)

The Magna RNA‐binding protein immunoprecipitation kit (Thermo) was used for the RIP assay. In brief, 1 μg of FBL and IgG antibodies were incubated with cell lysates overnight at 4°C. The protein A/G beads were incubated with cell lysates to capture RNA protein antibodies. RNA was extracted using TRIzol reagent and detected using qRT‐PCR.

### Statistical analysis

All data were analyzed using Graphpad Prism 7.0 software. All experiments were performed in triplicate and were analyzed using a two‐tailed unpaired student's *t*‐test. Data are presented as mean ± standard deviation. A *p*‐value of <0.05 was considered to indicate statistical significance.

## RESULTS

### Upregulation of FAM83A‐AS1 in LUAD tissues is correlated with poor patient prognosis

**FIGURE 1:**
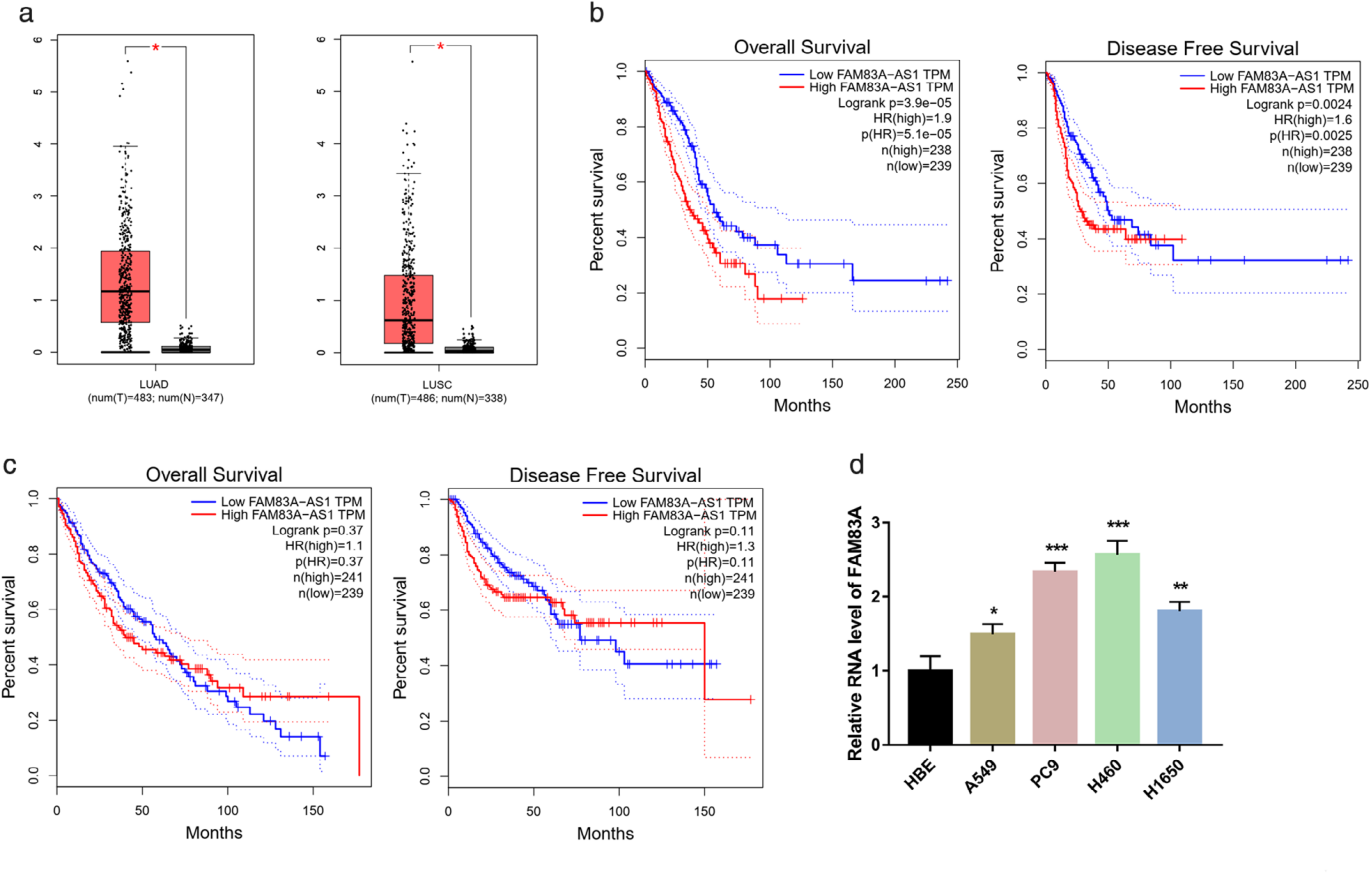
Upregulation of FAM83A‐AS1 in lung adenocarcinoma (LUAD) tissues is correlated with a poor prognosis. (a) FAM83A‐AS1 expression levels in LUAD and lung squamous cell carcinoma (LUSC) tissues and adjacent paracarcinoma tissue were determined using TCGA database. (b) The overall survival (OS) and progression‐free survival (PFS) of 477 LUAD patients with high and low FAM83A‐AS1 expression levels were analyzed. (c) The OS and PFS of 480 LUSC patients with high and low FAM83A‐AS1 expression levels were analyzed. (d) FAM83A‐AS1 expression was determined in non‐small cell lung cancer (NSCLC) cell lines and one human bronchial epithelial cell line, HBE. *p < 0.05; **p < 0.01; ***p < 0.001

Based on the TCGA database, the expression of FAM83A‐AS1 in LUAD and lung squamous cell carcinoma (LUSC) tissues was significantly higher than that in paracarcinoma tissues (Figure 1a). Moreover, the prognosis analysis of TCGA data indicated that the high expression of FAM83A‐AS1 in LUAD was closely associated with low overall survival (OS) and progression‐free survival (PFS), but FAM83A‐AS1 expression in LUSC was not correlated with either OS or PFS (Figure 1b,c). FAM83A‐AS1 expression was found to be consistently higher in NSCLC cell lines, compared with that in normal bronchial epithelial HBE cells (Figure 1d).

### FAM83A‐AS1 facilitated NSCLC cell growth and metastasis in vitro

**FIGURE 2:**
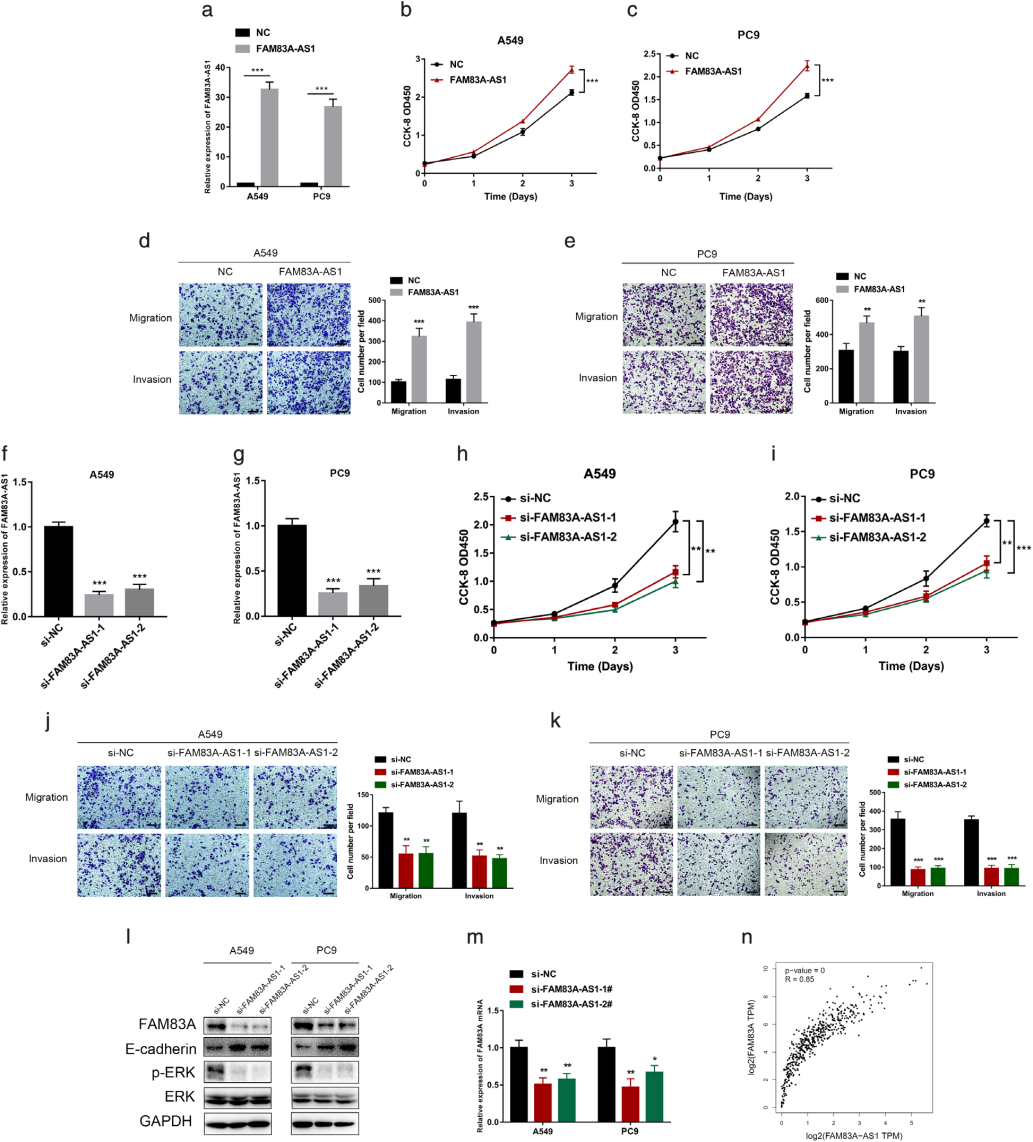
Downregulation of FAM83A‐AS1 inhibited lung adenocarcinoma (LUAD) cell growth and metastasis in vitro. (a) A549 and PC9 cells were transfected with an NC or pcDNA‐FAM83A‐AS1 plasmid. Quantitative reverse transcription PCR (qRT‐PCR) was then performed to detect the expression levels of FAM83A‐AS1. (b–c) CCK‐8 assay was performed to detect A549 and PC9 cell viability after the overexpression of FAM83A‐AS1. (d,e) Overexpression of FAM83A‐AS1 in A549 and PC9 cells and migration and invasion assays were used to detect the metastatic ability of the cells. (f,g) Two siRNAs were transfected into the A549 and PC9 cell lines, and qRT‐PCR was performed to detect the expression levels of FAM83A‐AS1. (h,i) CCK‐8 assay was performed to detect A549 and PC9 cell viability after the downregulation of FAM83A‐AS1. (j,k) Migration and invasion assays were conducted to detect cell metastatic ability. (l,m) The relative protein levels were measured using western blotting analysis. Then, qRT‐PCR was conducted to detect the expression levels of FAM83A. (n) The association between FAM83A‐AS1 and FAM83A expression in LUAD tissues was analyzed using Pearson correlation analysis. **p < 0.01; ***p < 0.001. Scale bar, 100 μm

We performed functional assays on two NSCLC cell lines, A549 and PC9, to explore the role of FAM83A‐AS1 in NSCLC cellular function. At first, we successfully overexpressed FAM83A‐AS1 in NSCLC cell lines (Figure 2a). CCK‐8 was performed to determine the viability of the cells. The results showed that FAM83A‐AS1 efficiently increased cell viability (Figure 2b,c). Then, a transwell assay was used to determine the migration and invasion abilities of the cells. The results indicated that FAM83A‐AS1 significantly promoted NSCLC cell migration and invasion (Figure 2d,e). We used two siRNAs to knockdown FAM83A‐AS1, and siRNA efficiency was detected using qRT‐PCR (Figure 2f,g). CCK‐8 and transwell assays showed that NSCLC cell viability (Figure 2h,i) and metastatic abilities (Figure 2j,k) decreased after FAM83A‐AS1 knockdown. WB showed that FAM83A‐AS1 knockdown also significantly decreased FAM83A and ERK expression levels in NSCLC cell lines (Figure 2l). This change suggested that FAM83A‐AS1 facilitates NSCLC cell growth and metastasis through its influence on FAM83A expression and ERK signaling pathways. Additionally, the downregulation of FAM83A‐AS1 decreased FAM83A mRNA expression (Figure 2m). The TCGA database was used to determine that FAM83A expression was positively correlated with FAM83A‐AS1 expression (Figure 2n).

### FAM83A promoted NSCLC via ERK signaling pathways

**FIGURE 3:**
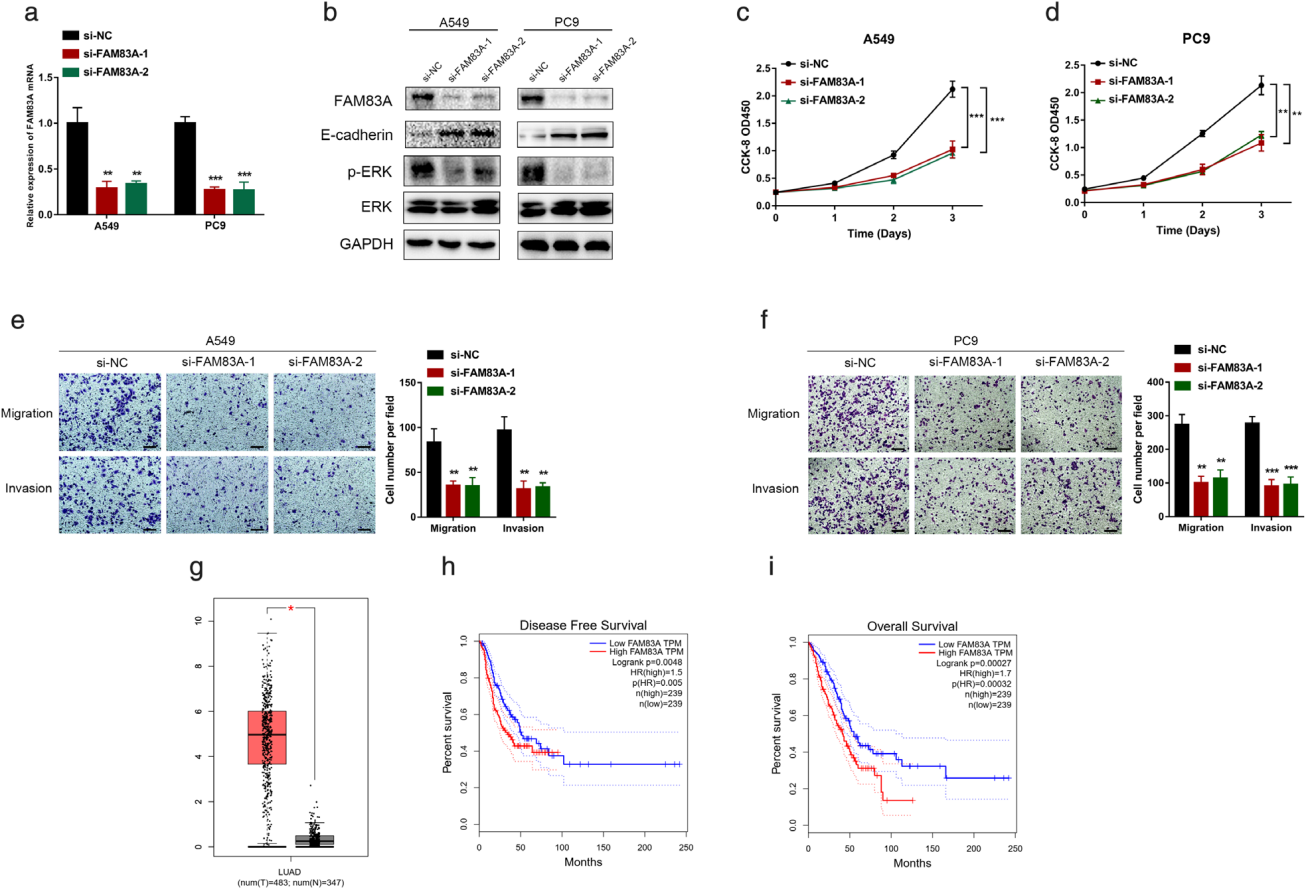
FAM83A exerts its oncogenic functions in LUAD. (a,b) FAM83A expression was knocked‐down using two siRNAs, and quantitative reverse transcription PCR (qRT‐PCR) was performed to detect the FAM83A expression levels. CCK‐8 assay (c–d), migration and invasion assays (e–f), and western blotting analysis were performed to detect FAM83A expression in A549 and PC9 cells treated with the siRNAs. (g) FAM83A mRNA expression in LUAD was obtained from TCGA database. (h,i) The OS and PFS of the 478 LUAD patients with high and low FAM83A expression levels were analyzed. *p < 0.05; **p < 0.01; ***p < 0.001. Scale bar, 100 μm

FAM83A mRNA expression was detected using qRT‐PCR after FAM83A‐AS1 knockdown to explore the relationship between FAM83A‐AS1 and FAM83A. The results indicated that FAM83A mRNA expression decreased following the decrease in FAM83A‐AS1 expression. We used two siRNAs to knockdown FAM83A to determine whether FAM83A‐AS1 required FAM83A to perform its functions. qRT‐PCR and WB showed FAM83A was knocked down by siRNAs (Figure 3a,b). Furthermore, E‐cad and p‐ERK expression also decreased, indicating that FAM83A expression was correlated with the ERK signaling pathway (Figure 3b). Next, CCK‐8 assay and transwell assay were performed, and the results showed that the downregulation of FAM83A significantly inhibited NSCLC cell viability (Figure 3c,d) and metastatic ability (Figure 3e,f). TCGA database showed that FAM83A expression at mRNA level was remarkably higher in LUAD tissues than that of paracarcinoma tissues (Figure 3g). Moreover, FAM83A expression in LUSC was closely associated with OS and PFS (Figure 3h,i).

### FAM83A‐AS1 increased FAM83A expression by enhancing pre‐mRNA stability

**FIGURE 4:**
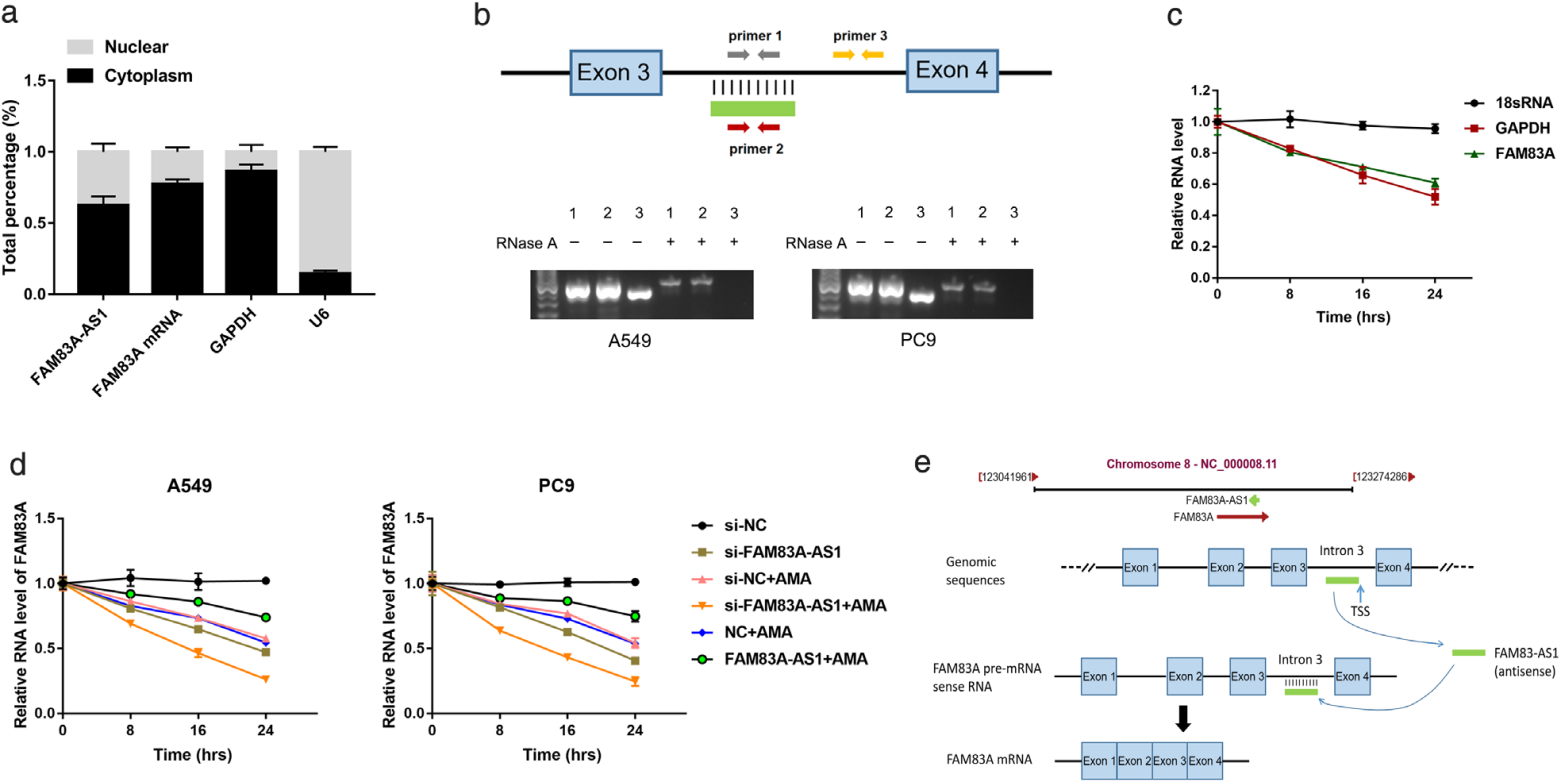
FAM83A‐AS1 increased FAM83A expression by enhancing pre‐mRNA stability. (a) FAM83A‐AS1 and FAM83A mRNA expression in the cytoplasmic and nuclear fractions were detected using quantitative reverse transcription PCR (qRT‐PCR). GAPDH and U6 served as positive controls. (b) PCR was conducted after RNase protection analysis (RPA). (c) Stability of FAM83A and GAPDH transcript was measured relative to 0 h after treated with AMA in A549. The 18S gene acted as a reference gene. (d) qRT‐PCR analysis of FAM83A expression in LUAD cells with FAM83A‐AS1 knockdown/overexpression and AMA treatment for a specific duration. The 18S gene acted as a reference gene. (e) The pattern of FAM83A‐AS1 bound to the intron of FAM83A pre‐mRNA and evidence of enhanced FAM83A mRNA stability. *p < 0.05; **p < 0.01; ***p < 0.001

Since FAM83A‐AS1 influenced FAM83A expression, we needed to clarify the mechanism by which FAM83A‐AS1 regulated FAM83A. Subcellular fractionation assay showed that approximately 40% of FAM83A‐AS1 was located in the nucleus, while 60% was located in the cytoplasm (Figure 4a).

Antisense transcripts may regulate the stability of the sense transcripts by interacting with them to form double‐stranded RNA structures.^14^ FAM83A‐AS1 and FAM83A are transcribed at the same DNA location but in opposite directions. The entire strand of FAM83A‐AS1 overlaps within the third intron of FAM83A (Figure 4b). RNase protection assay was used to detect the RNA duplex structure between FAM83A‐AS1 and FAM83A. The RNase A enzyme digested the single‐stranded RNA but did not digest the double‐stranded RNA. The presence of the RNA overlapping duplex was confirmed using RT‐PCR (Figure 4c). RNA–RNA duplex can protect RNA from RNase degradation and increase its stability by changing the secondary or tertiary structure of RNA.^15^, ^16^ We used an RNA polymerase II inhibitor (α‐amanitin) to assess the half‐life of FAM83A‐AS1, FAM83A, GAPDH, and 18 s RNA to determine whether the duplex structure between FAM83A‐AS1 and FAM83A influenced FAM83A mRNA stability. The results showed that the upregulation of FAM83A‐AS1 increased the stability of FAM83A, and conversely, the downregulation of FAM83A‐AS1 decreased the stability of FAM83A (Figure 4d). These results confirmed that FAM83A‐AS1 enhanced FAM83A stability by forming an RNA duplex with FAM83A pre‐mRNA (Figure 4e).

**FIGURE 5:**
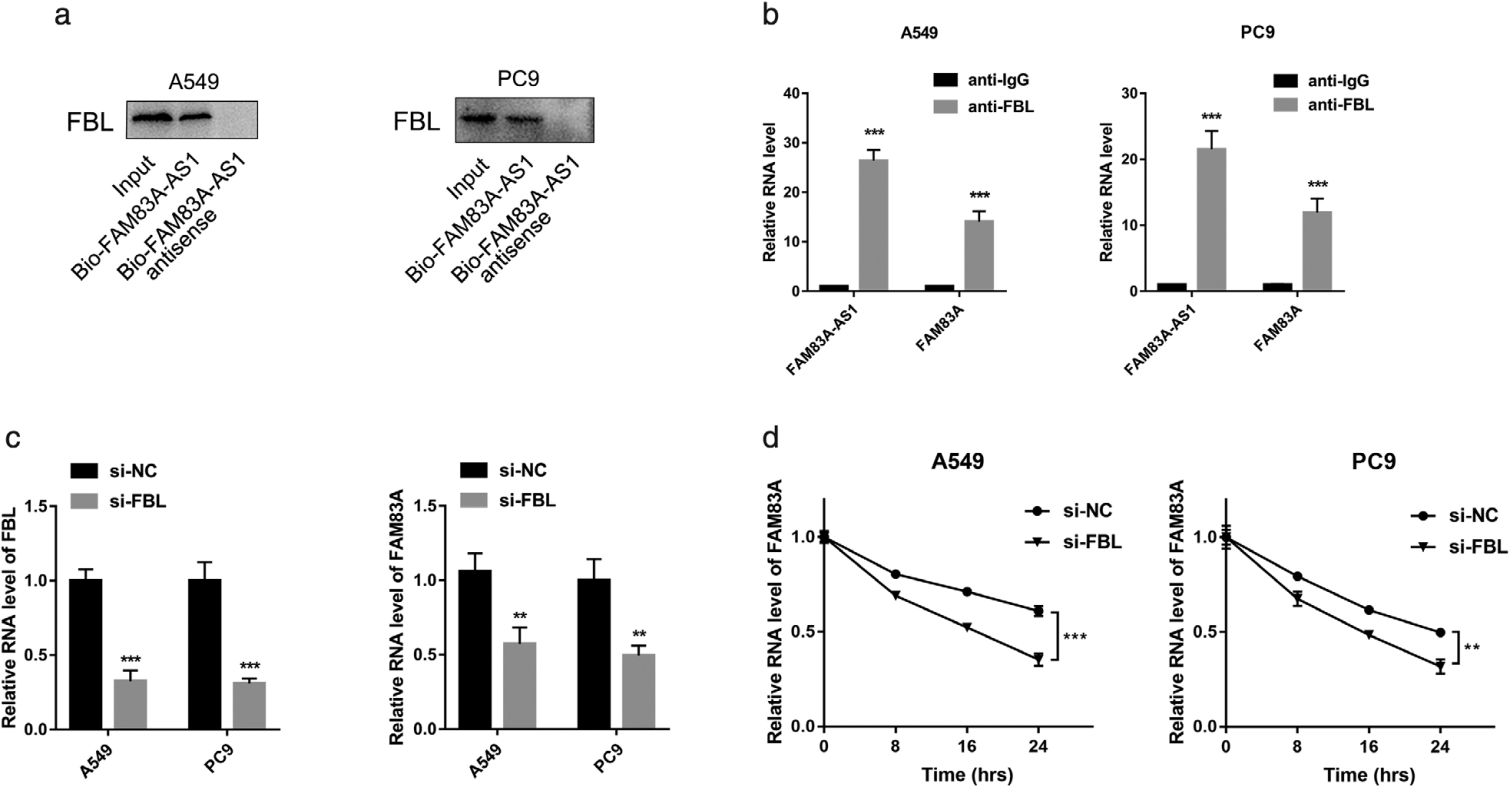
FAM83A‐AS1 enhanced FAM83A mRNA stability by binding to FBL. (a) RNA pulldown and western blotting analysis were used to determine the binding relationship between FAM83A‐AS1 and FBL. (b) RIP assay was performed on LUAD cells using IgG and FBL antibodies. (c) Quantitative reverse transcription PCR (qRT‐PCR) analysis of FAM83A expression in lung adenocarcinoma (LUAD) cells treated with FBL siRNA. (d) qRT‐PCR analysis of FAM83A expression in LUAD cells with FBL knockdown and AMA treatment for a specific duration. The 18S gene acted as a reference gene. *p < 0.05; **p < 0.01; ***p < 0.001

The results of the RNA pulldown assay indicated that FAM83A‐AS1 could bind with FBL (Figure 5a). Subsequently, RIP assay showed both FAM83A‐AS1 and FAM83A were enriched by anti‐FBL (Figure 5b), indicating that FAM83A‐AS1 could bind to FBL. After the knockdown of FBL, qRT‐PCR was performed to confirm the relationship between FAM83A and FBL. The results showed that FAM83A expression decreased when that of FBL decreased (Figure 5c). Moreover, FAM83A mRNA stability decreased upon FBL depletion (Figure 5e). Together, these results demonstrated that FAM83A‐AS1 enhanced FAM83A mRNA stability not only by forming an RNA duplex but also by binding with FBL (Figure 5f).

## DISCUSSION

We demonstrated a novel molecular mechanism by which FAM83A‐AS1 positively regulated FAM83A. Many studies have shown that lncRNAs play a critical role in the progression of diseases, including cancer.^17^, ^18^ For instance, Wang et al. reported that high lncRNA HOXA11‐AS expression was correlated with the poor prognosis of glioma patients.^19^ Linc00312 expression was negatively correlated with tumor size and the occurrence and progression of NSCLC.^20^ In this study, FAM83A‐AS1 was significantly upregulated in NSCLC, and the high expression of FAM83A‐AS1 in LUAD tissues was closely associated with OS and PFS.

Previous evidence has shown that many protein‐coding mRNAs have natural antisense transcripts and that these non‐coding RNAs account for most of the length of each mRNA.^21^, ^22^ Natural antisense transcript RNAs (NATs) are closely associated with the occurrence and development of cancer. For instance, NKX2‐1‐AS1 upregulates oncogene NKX2‐1 and promotes the proliferation of lung cancer cells.^23^ DHPS, as a NAT of WDR83, plays a vital role in the tumorigenesis of gastric cancer (GC).^16^ Shi et al. found that FAM83A‐AS1 upregulates and promotes NSCLC cell proliferation and invasion.^24^ Similar results were obtained through this study. The overexpression of FAM83A‐AS1 promoted NSCLC cell proliferation and metastasis, while FAM83A‐AS1 knockdown produced opposite results. Together, these findings reveal that FAM83A‐AS1 acts as an oncogene for the tumorigenesis of NSCLC.

NATs are involved in several mechanisms, including the regulation of the sense gene expression and stabilization of the sense transcript. Beltran et al. found that ZEB2‐AS1 promotes ZEB2 protein expression, which further inhibits E‐cadherin expression. Similarly, PTB‐AS promotes gliomagenesis through the increase in PTBP1 levels by directly binding to PTBP1 3′UTR and stabilizing its mRNA.^25^ HAS2‐AS1 and HAS2 are transcribed from a bidirectional promoter, and HAS2‐AS1 stabilizes HAS2 mRNA expression by forming an RNA/mRNA heteroduplex.^26^ In this study, we identified a natural antisense transcript, FAM83A‐AS1, which originates from the intergenic regions of the FAM83A gene. We also found that FAM83A‐AS1 formed a duplex structure with the FAM83A pre‐mRNA and increased FAM83A expression by enhancing the stability of the FAM83A‐AS1 pre‐mRNA. On the contrary, the downregulation of FAM83A‐AS1 decreased FAM83A expression.

RNA‐binding proteins (RBPs) can directly bind to RNAs and play essential roles in mRNA stabilization, splicing, translation, and localization.^27^ Previous studies have shown that several RBPs are involved in the initiation and progression of malignancies.^28^, ^29^ Eukaryotic initiation factor 4A3 (eIF4A3) induces circMMP9 expression by binding to MMP9 pre‐mRNA and also promotes glioblastoma multiform proliferation and metastasis.^30^ The RNA binding protein, RBM3, promotes the stemness of colorectal cancer cells by enhancing β‐catenin activity.^31^ He et al. found that FAM83A‐AS1 enhances FAM83A mRNA stability by binding with NOP58.^10^

We also found that RBP FBL could bind to FAM83A‐AS1 and FAM83A to increase FAM83A expression. We speculated that FAM83A‐AS1 binds to FAM83A pre‐mRNA by forming an RNA/mRNA heteroduplex and that RBP FBL binds to the duplex to enhance its stability.

In summary, we found that FAM83A‐AS1 was upregulated in NSCLC and was associated with a poor outcome. We also demonstrated that FAM83A‐AS1 promoted NSCLC proliferation and metastasis by increasing FAM83A expression. Overall, this research study demonstrated a novel oncogenic pathway in NSCLC tumorigenesis. Our findings may shed new light on the molecular mechanisms by which NATs modulate NSCLC tumorigenesis.

## CONFLICT OF INTEREST

No potential conflicts of interest were disclosed.
